# Traumatic journeys; understanding the rhetoric of patients’ complaints

**DOI:** 10.1186/s12913-018-3339-8

**Published:** 2018-07-16

**Authors:** May McCreaddie, Bethan Benwell, Alice Gritti

**Affiliations:** 1 School of Nursing and Midwifery, Royal College of Surgeons in Ireland, Medical University of Bahrain, PO Box 15503, Adliya, Kingdom of Bahrain; 2 Senior Lecturer in English Language and Linguistics, Faculty of Arts and Humanities, University of Stirling, Scotland, UK; 3 Newbattle Abbey College, Scotland, UK

**Keywords:** Healthcare complaints, Rhetoric, Discourse analysis, Psychological distress, Grievances

## Abstract

**Background:**

Research on patients’ complaints about healthcare has tended to focus on the typology of complaints and complainants to homogenise complaints and better understand safety implications. Nonetheless, complaints speak to a broader spectrum of harm and suffering that go beyond formal adverse events. Complaints about care episodes can take considerable time and effort, generate negative energy and may leave a dogged ‘minority’ embittered.

**Methods:**

This study provides an overview of the process and rhetoric of how patients formulate written complaints. We collated a data corpus comprising 60 letters of complaints and their responses over a period of one month. This paper focuses on the complaint letters only. National Health Service (NHS) Complaint Department staff in a healthcare area in the United Kingdom (UK) anonymized the letters. We took a broad qualitative approach to analysing the data drawing upon Discourse Analysis focusing on the rhetorical and persuasive strategies employed by the complainants.

**Results:**

What patients complained about related to how they complained, with complainants expending considerable effort in persuasive rhetoric that sought to legitimise the complaint drawing upon different sources of epistemic authority. The complainants struggle to be an ‘objective’ witness as the complaint evolves from an implicit neglect narrative to increasing ‘noise’ with other features such as Direct Reported Speech used to animate and authenticate the narrative.

Many of the complex complaints appeared to evidence some psychological distress. This was associated with the complainants’ reports of experiencing cumulative poor health care and their repeated failure to resolve the complaint. The subsequent delicate and potentially stigmatized formal act of complaining was a source of additional distress.

**Conclusions:**

Complaints are involved narratives often predicated on the expectation they will not be given due credence. Health care staff may benefit from understanding how complaints are formulated to be able to more appropriately address the focus and extent of patients’ grievances from the outset and therefore, reduce the considerable associated harm.

## Background

A complaint is a problem - a gap between ‘customer’ expectation and receipt of a product or service [1]. Consumer industries posit complaints as an opportunity to retain loyal customers, improve service and expand [7]. However, public service providers, specifically the National Health Service in the United Kingdom (NHS) and its workforce, have an arguably different synergy with their consumers.

Eighty-three per cent of health care in the UK is publicly funded and hence, customer loyalty is largely unchallenged [25]. Further, NHS consumers are emotionally invested in, and custodial guardians of, ‘their’ NHS [34] holding concomitant high expectations of healthcare [30] exemplified by the most common reason for complaining about the NHS: to prevent a similar distressing event from happening again to others [16, 51, 55]. Despite NHS consumers’ custodial expectations, the Francis Report [13] uncovered a litany of failings in one NHS trust in England and specifically highlighted complaint management as exacerbating justified grievances and undermining public trust. Complaints are therefore, a pervasive feature of modern healthcare and personify the pre-eminent role of the patient-consumer relationship.

UK NHS complaints data has primarily been used from a safety and quality lens to highlight the patient perspective using reliable data not captured elsewhere e.g. [28, 58]. Consequently, how best to organize this data and interpret its portent, including its predictive value, has been the focus of much debate. A qualitative study by Kroening et al. [27] suggested 2 out of 52 high level incidents could have been predicted from complaints data whilst medical staff at greater risk of complaints have also been identified [4, 56]. Complaints data may therefore, be a partial indicator of varying degrees of harm and quality but it remains a diverse spectrum of unrepresentative data [44]. Complaints are therefore, not homogenized data but subjective and individual events that are subsequently aggregated to provide an overview of problematic interactions, areas or individuals. Nevertheless, Gallagher and Mazor [17] suggest that complaints – as individual instances of (actual or perceived) harm – should be deemed adverse events and accorded due status.

Complaints research thus far has therefore, tended to focus on the typology of complaints and complainants (e.g [4, 6, 44]) – outlining the characteristics of complainers and attempting to objectify the focus of their grievance. Notably, Gillespie and Reader [18] developed a tool for coding complaints in terms of severity with the view that a non-subjective typology of complaints would hopefully lead to better response times, management and prevention of harm. Nevertheless, there is arguably a need to delineate (homogenized) complaints as possible sentinel events representative of system failure or general predictors of safety-related harm, from heterogeneous, individual, legitimate grievances that encapsulate a broader sense of ‘harm’. The former may prompt medico-legal redress. However, the latter abrogates the notion of patient-centredness and instead of harnessing the NHS’s greatest allies – patients and relatives – potentially creates a reservoir of embittered custodians whose individual concerns and grievances have not been appropriately resolved.

We know that NHS complaints increase year on year [50], predominantly involve medical and nursing staff, are invariably multi-factorial and most likely to focus on care and treatment, specifically communication or interpersonal aspects of healthcare [19, 46]. Moreover, approximately one third of these complaints will not be upheld, having been investigated by the self-same body against whom the complaint was made [42]. Nonetheless, of all referrals to the Ombudsman (SPSO) - the next level of complaint escalation – over half are subsequently upheld [49]. Thus, local attempts at resolving written complaints are largely ineffectual and may exacerbate the complainants’ sense of grievance. There is therefore, evidently a need to explore in more detail, *how* people complain in order to better assess, address and remedy their grievance at the earliest opportunity.

We believe it is important to better understand why and how – in the broadest sense – patients and their relatives formulate complaints. Accordingly, we believe it is crucial, given the possible misunderstandings that may have led to the complaint in the first instance, to consider whether the written dialogue between complainant and complaint handler is fully explicated. A fine-grained linguistic analysis can provide both an overview of the process and actions taking place and an enhanced understanding of the nuances of the rhetoric being employed. Complainants are frequently dissatisfied, not only with the outcome of their complaint, but also the formulation of the response [13, 44, 60]. If complaint handlers can learn from a detailed appreciation of the emotional labour that goes into formulating complaints, this may contribute positively to a more sensitive and empathic approach to complaints handling.

This paper provides an overview of complainants and their complaints and the way in which those individual grievances are expressed. A further paper will review the complaint responses.

## Methods

We took a broad qualitative approach drawing upon Discourse Analysis (DA). Discourse Analysis is the study of text and talk and focuses on how speakers – or in this case – writers’ attempt to present, persuade and perform or accomplish certain actions. The focus is therefore, on what is happening (action) rather than cognition (understanding) or behaviour. The participants do attribution; they infer the causes of events or behaviours. Thus, reports and descriptions are displayed as fact by a variety of discursive devices and are rhetorically organised to undermine alternatives. We therefore, largely drew upon the Discursive Action Model [12] which is based upon insights from Conversational Analysis and Discursive Psychology more generally [20, 24, 41].

Our data corpus comprised a purposive sample of written complaints and their responses collated over the period of one month. The analysis presented here is based on an overview of patternings of all 50 complaints, with examples extracted from 31 and fine-grained and systematic qualitative analysis focusing on 2.

Preliminary analysis of the data started through repeated reading of the complaint letters, asking questions of the data (such as ‘why this utterance/phrase/action now?’) and making analytic notes. All data was digitised and entered into a password-protected database. The data was coded and specific features identified (e.g. complainer relationship, type, focus, purpose of complaint, length, outcome) with structure, themes and discursive fragments or features tagged and reviewed. Data was analysed by applying sequential and systematic observation of particular discourse strategies or linguistic/textual choices from initial coding through to linguistic and rhetorical practices. Particular segments of the data were subject to an enhanced linguistic analysis for specific discursive features e.g. extreme case formulations (ECFs), idioms, punctuation, reported speech - and compared with their function in previous studies. We also reviewed and discussed data segments with colleagues at the SEDIT Group (Scottish Ethnomethodology, Discourse, Interaction & Talk) who are experienced in the use of DA. The data corpus and the analytical approach allows for a thorough review of the rhetorical and persuasive strategies employed by the complainants.

### Ethics

Ethical approval was obtained from the local Research Ethics Committee (11/ES/0048). The complaints department staff collated all written complaints and their responses over the period of one month. This data was fully anonymised by complaints department staff prior to being received by the researchers. This negated the need for explicit a priori consent. We completed the Caldicott data protection consent form [57]. Data was securely held in storage (soft and hard copies) as per Economic and Social Research Council guidelines [10].

### Data corpus

Our data corpus comprised 60 letters in total with 49 paired written complaints: complaint and response - plus 10 response letters and one complaint letter with no response (Table 1). Of the 50 complaints, it was only possible to specify gender in 43 with females being the main complainers (*n* = 27). Females were more likely to complain as a next of kin (NOK) (20 out of 28 NOK) than as a patient (8 out of 22) with Mothers and Daughters the main complainers on behalf of the NOK (15 out of 20/28). Ten of the 50 complaints were made on behalf of patients by a third party/advocate (Member of Scottish Parliament *n* = 9, Citizens Advice Bureau *n* = 1) with 7 of these being initiated by male patients.

**Table 1:** Data corpus

Letter Category	Number	C&T	A
Paired Complaint: Complaint and Response	49	37	12
Complaint only	1		1
Complaint response only	10	5	5
TOTAL	60	42	18

*C&T* care and treatment, *A* administrative

The vast majority of complaints were typed or emailed (40/50), three were written on comments/feedback sheets, with two written by a complaint handler (CH) via a telephone complaint. The remaining 6 were handwritten including one in capitals throughout and one other in calligraphy. The vast majority of complaints were one page long with the longest four pages. Replies tended to be double the length of the complaint with the longest being eight pages.

#### Nature of the complaints (*n* = 60)

The complaints were placed into two broad categories. Complex complaints were care and treatment-related (*n* = 42) involving at least two or more issues, usually over a given time period. Single issue complaints outlined administrative issues (*n* = 18). These categories depicted the degree of complexity regarding the complaint and its primary focus.

Care and treatment is an admittedly broad category that encompasses human interactions (communication, personal attributes) fundamental care e.g. washing, dressing and, procedures. Certain aspects of care e.g. human interactions are inextricably linked with fundamental care and or procedures and hence it was impossible to separate these aspects. In addition, once moved to formally complain, complainants usually identified several issues of grievance. Consequently, care and treatment complaints tended to be fairly complex, involving a chronological outline of events over time. For example, 12 of these complaints were fairly convoluted depicting long sequences of care and treatment with 4 involving the death of a loved one. Conversely, administrative complaints were single issue protests such as those dealing with the operational, functional aspects of the health service such as waiting times, referrals, letters, delays and of course, car parking.

We will now outline the findings.

## Results

The nature of the complaint was inextricably linked to how it was expressed. For example, single issue complaints were simple, brief and focused. These single administrative complaints were relatively straightforward and posed little or no threat to patient safety or harm. Conversely, care and treatment complaints were often complex, involving sequences of care with substantial accompanying detail. These complaints may or may not have posed a threat to patient safety but certainly caused the complainant or their advocate some degree of harm such as psychological distress. We will therefore, largely focus on the latter category as (a) they constitute the majority of the complaints and (b) often failed to elicit a positive or preferred outcome.

### Building the case

**Fig. 1:**
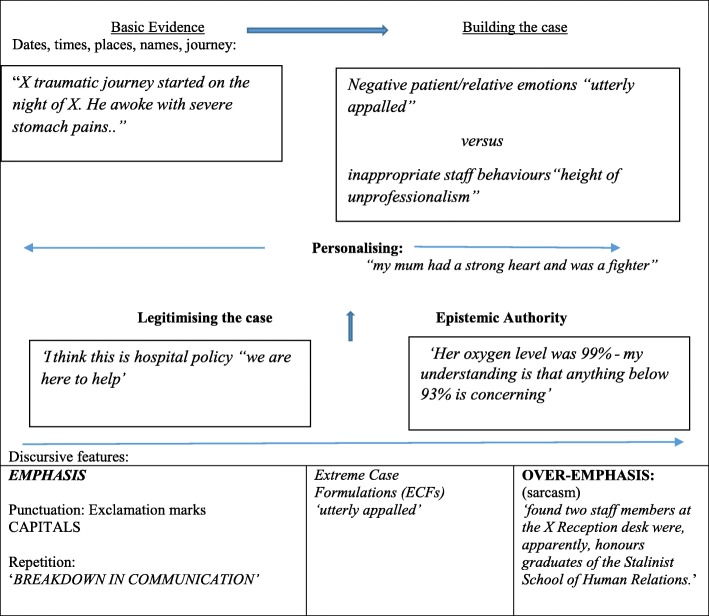
An overview of the rhetorical and persuasive strategies used by complainants

Complainants focussed on the action or process of events and provided reports and descriptions displayed as fact. These reports were underpinned by a variety of discursive devices and were rhetorically organised to undermine alternatives. In so doing they built a case of ‘evidence’ that sought to weaken any competing reality or alternative view. An overview of the rhetorical and persuasive strategies employed by the complainants is provided in Fig. 1 and reviewed in detail below.

The complaints demonstrated a strong narrative of implicit inferred neglect that evolved from a relatively simple, polite, formal and largely objective overview of the problem (narrative-objective) to a more subjective, explicit opinion of events (narrative-subjective). Initially and sometimes briefly, the complainant offers a simple empirical account of events that offers pertinent information (existing medical condition, presenting medical condition, care status etc.) in a chronological fashion (dates, times, places) providing an initial context for the complaint. The information is outlined succinctly, politely and formally. Additionally, the complainant provides simple but illustrative and implicit descriptions of events. This initial groundwork gives way to a more assertive and subjective representation of events involving contrastive work (good versus bad) and active voicing e.g. ‘if you get her in a home she’ll have her own room and TV (Television)’. Notably, this shift from implicit to explicit complaining appeared to occur when a perceptible complaint threshold was breached by (a) the complainants’ accounts of perceived, repeated and cumulative grievances (e.g. an appointment being cancelled four times in succession) and, or (b) when a specific and serious (final) incident occurred.

The complainant may also bridge the move from implicit to explicit by personalizing the issue at hand, thereby authenticating the grievance. Accordingly, the initial bold descriptive ‘facts’ are given a human face emphasizing the important, intimate and life changing nature of the service provided and therefore, the perceived harm accrued. Thereafter, these now personalized ‘facts’ seamlessly merge into detailed discursive work that contrasts the now competing protagonists and their respective perceptions and behaviours.

Two rhetorical and persuasive strategies; ‘legitimising the complaint’ and ‘epistemic authority – corroborating’ - scaffold the overall aim of ‘building the case’ and appear to have two discrete functions. In ‘legitimising the complaint’ complainants’ seek to further justify and assert their right to complain as reasonable socially-aware individuals. This is achieved in various ways. First, the complainant seeks to establish themselves as someone who observes and upholds rules, who has a (reasonable) expectation of help and is cognizant of competing demands (time, workload, other patients). Second, some complainants demonstrated a social conscience about this publicly-funded service i.e. use of resources, time. Finally, a number of complainants underpinned these reasonable expectations with an apparent reluctance to complain. Nevertheless, they reported that the number of accumulating grievances and the subsequent scale of the perceived injustice necessitated a formal complaint.

The zenith of the complainants’ ‘case’ is the use of epistemic authority to provide further corroboration by asserting formal or lay knowledge of a specific medical condition or intervention. In a smaller number of cases this resulted in a subsequent accusation of negligence. Particular discursive features (punctuation; capitalization, exclamation mark) were manifest throughout and tended to be most evident when illustrating and contrasting perspectives and behaviours, therefore, emphasising the evidence and the perceived harm. This occasionally resulted in the use of superiority humour, specifically sarcasm e.g. “can the X left hand be introduced to the NHS right hand?” [3970]. Accordingly, these persistent discursive features enhanced epistemic authority and the nature of the rhetoric being employed.

### Narrative-objective: Describing

The rhetorical persuasiveness, the degree of context provided and the accompanying level of detail were evident from the outset of the complaints. Complainants patently wished their grievances to be viewed holistically so their ordeal could be better understood, their objections justified and the associated harm laid bare.

The complainant initially sets out their case by describing objective details in a chronological fashion including dates, times, names and places. This initial ‘evidence’ was largely a descriptive narrative account of events as they started to unfold as in *“I am writing to acquaint you with the facts” [3945]* - and appeared to be largely objective. However, even at this early stage, the complainant appears to be working to convince the reader of the scale of the grievance(es) and hence, we witness the first use of extreme case formulations (ECFs; see [40]);

*Not welcoming in any way [3923]*

*Completely unacceptable [3926]*

*I have no faith [3945]*

ECFs invoke minimal or maximal properties of events and are used to defend complaints, claim objectivity and, or infer right or wrong [59]. The expression of discontent at this juncture was usually proportional to the complexity and length of the complaint i.e. the more complex complaints tended to generate more ECFs. Moreover, the complainant’s displeasure was invariably evident throughout the letter and not simply restricted to the introduction or conclusion of the complaint, becoming increasingly embedded in a personal affective and behavioural discourse as momentum built. Thus, the complaint follows a narrative timeline or ‘journey’ befitting the intimate and delicate nature of the supposed wrongdoing. The following examples establish the complainants’ perceptions of their reported experiences as some kind of odyssey and also signal the start of the inferred neglect narrative;

*1. X traumatic journey*

*2. started on the night of X*

*3. when he awoke with severe stomach pains [3932]*

*1. …My daughter*

*2. was sent out of the hospital*

*3. in her pyjamas and housecoat with trainers on*

*4. in [sic] a very windy, snowy morning and the ground was slippery. [3948]*

In the first account [3932] a father is complaining about his young son’s care. He provides factual information from the outset (date; night of X) and establishes the perceived seriousness of the situation with an ECF (severe stomach pains) that caused his son to wake during the night. ECFs are often used when complainants might expect their claim to be undermined and hence, there is a need to portray their case in such a way as to justify their actions. Thus, the seriousness of the son’s condition and the Father’s accompanying and justifiable concern needed to be established from the outset. What is arguably different in this extract is the use of ‘traumatic journey’ which is not neutral. Most complainants outlined their experiences in a relatively low-key temporal and objective format with this building to a robust account. Thus, the complainants recounted and relived their perceived distress in print in a further effort to obtain some resolution and arguably attempted to do so in a restrained and objective way.

In 3948, a mother complains about her daughter’s care and personalizes the complaint from the outset (1; my daughter). Lines 2 to 4 is a good example of an inferred neglect narrative, in that the daughter was not just discharged but ‘sent out’ rather than presumably permitted or encouraged to wait in the warmth of the hospital. Simple descriptions are being provided, but they are carefully constructed, contrasting and implicit, building a picture that allows the reader to draw their own unequivocal conclusions, with ECFs also evident at the outset (e.g. very windy). Thus, Line 3 (pyjamas, housecoat, trainers) are patently inappropriate clothing for the weather conditions reported at the time (windy, snowy, slippery) putting the daughter at risk of hypothermia or a fall, in addition to any mental trauma accrued from her reported untimely and inappropriate banishment. The simple but damning detail provided by the complainants describe as objectively as possible their experiences, yet it is anything but neutral.

The following example further evidences the inferred neglect narrative that evolves into a narrative-subjective account;

*1. I arrived at visiting time*

*2. and found my father*

*3. in a single patient room*

*4. wearing a thin t-shirt the window was open,*

*5. the room was very cold, no heating was on and*

*6. he had not been given the buzzer for contacting a nurse [A: 3961]*

The above account [3961] is of a daughter complaining on behalf of her 74-year old father who has end-stage Motor Neurone Disease which has rendered him aphasic (speechless) and completely dependent upon others. He has been admitted with vomiting and diarrhea and aspiration pneumonia - facts and context that are briefly provided at the start of the letter. The above is the opening sequence of the complaint. The daughter opens the initial complaint sequence with the information that she arrives ‘at visiting time’ and is therefore, presumably congruent and observant of the routine and rules of the institution. In line 2, she states her recollection that she had to actively seek out her father’s whereabouts on her own. i.e., − she was not met by nursing staff and directed to her father’s room. The use of ‘my father’ is formal and polite but also potentially personalizes any perceived harm on account of the relationship. Notably, as the complaint subsequently becomes more subjective, the daughter uses the more informal ‘dad’. However, it is in line 3 that the inferred neglect narrative commences in full. Thus, despite the preamble illustrating her father’s vulnerabilities, he has been placed in a single room, alone. Line 4 is objective, empirical and illustrative, merging into a slightly more subjective-objective and contrasting account in line 5. In line 6 the inferred neglect narrative becomes more explicit as this graphic picture of a vulnerable patient, sitting ill and alone in a single room without the means for summoning help, is laid bare. As such, the daughter outlines fundamental lay expectations of care that have gone unaddressed by professional staff entrusted with the welfare of her vulnerable father. The picture of inferred neglect is further corroborated by her father;

*1. He communicates through an ipad.*

*2. And he told me that no-one.*

*3. Had been in the room.*

*4. Since he was transferred [B:3961].*

### Narrative – Subjective: Illustrating and contrasting

The initial neutral, objective and inferred groundwork of the complaint inevitably gives way to a more robust representation of events with the narrative not just describing but illustrating and contrasting. Some complainants might dispense with the formality and neutrality or the opening sequence and reach this more subjective sequence quicker than others.

The following is a sequence from a complaint by a granddaughter about the general care and treatment of her elderly grandmother in a care of the elderly unit. Notably, this section of the complaint is prefaced by a list of initial concerns. The complainant states that her, her mother and aunt, had resisted in making these concerns formal until the following incident took place and the complaint threshold was breached. In short, the grandmother was unable to see the ward television due to her position in the unit. Accordingly, the mother and aunt had apparently looked in on the other units for comparison and were allegedly noticed in so doing by a staff nurse (X) who ‘*asked them what they were doing’*.

*1. My mother explained and X then proceeded to respond in an unprofessional manner*

*2. in fact returning to my grans room and in a very loud voice (waking a sleeping patient)*

*3. made several comments including but not limited to:*

*4. It’s not my fault – I didn’t build the ward*

*5. You’re saying we’re singling your mother out (it should be noted this was never said or implied by my mother or aunt)*

*6. If you get her in a home she’ll have her own room and TV.*

*7. During the response, my aunt raised her concerns of such an unprofessional conversation taking place in front of and disturbing patients [3950]*

First, the complainant starts the sequence with a personal, polite, formal and reasonable ‘my mother explained’ i.e. the mother sought understanding. However, despite this apparently restrained opening, the complainant does not simply state ‘X responded’ or even ‘X then responded’ but rather ‘X then proceeded to respond’. Thus, the subsequent reporting of X’s inappropriate behaviour is enhanced by the preceding protracted and sequential use of ‘proceeded to respond’ that perhaps enhances a slightly more formal account. Second, the complainant then names her perception of the behaviour exhibited by X (unprofessional manner) with supposedly factual information (returning to my grans room) with some observations (in a very loud voice) supported by further empirical evidence (waking a sleeping patient): all aspects working to corroborate her testimony. These details provide empirical validation of the complaint with the waking of the second patient both a secondary complaint and evidence of the consequences of the nurse’s ‘unprofessionalism’. Third, the apparent active voicing of three damning statements is also notably prefaced by ‘made several comments including but not limited to’. Thus, the complainant is indicating that the complained-about party made more than one and possibly more than two comments, subsequently providing three discrete examples. Accordingly, the complainant is arguably demonstrating further restraint and reason in the face of considerable provocation. Notably, the phrase ‘including but not limited to’ is more in keeping with a formal document such as the listing of duties on a job description and as such, it perhaps serves to highlight further the inappropriate nature of the alleged statements. Moreover, the reported speech is not provided in quotation marks but as a simple list.

The absence of the quotation marks in this reported speech segment is at odds with the fairly precise punctuation elsewhere in the sequence or indeed, throughout the complaint letter. The lack of speech marks here may also serve to enhance the rational account being provided as perhaps the use of appropriate punctuation here may have drawn further attention to the three statements and have caused the reader to doubt their veracity as reported speech. Moreover, it is arguably reasonable to assume someone can remember one phrase or sentence verbatim but perhaps not three.

Reported speech is often used to suggest distance from a proposition (see [29]) but it is also a way of empirically recreating an account, thereby damning the original speaker through reporting of their (unreasonable) words (e.g. [54]). However, in the above sequence the complainant identifies a form of reported speech by the nurse in question (L5) ‘you’re saying that’ which she firmly rejects as an aside in parenthesis thereby enhancing her own account and further damning the nurse.

Finally, the last line in this sequence is interesting in that it again, perhaps infers some neutrality or distance on the part of the complainant with ‘the response’ and not ‘her’ response. ‘The response’ is clearly that of the healthcare worker which the aunt presumably interrupts or interjects as she states ‘during’. However, this is importantly not stated by the complainant as it may serve to undermine her case, or indeed it may simply be that she is seeking to stop the healthcare workers from disclosing personal information about the grandmother. The concluding lines (L6, L7) in this sequence are noteworthy in that they return to the polite formality and inferred reasonableness of the opening line (L1) naming inappropriate behaviour but at the same time minimising or controlling the aunt’s disquiet via ‘raised her concerns.’ Further admonishment of the behaviour via the complainant’s empirical corroborating observations completes the sequence.

Throughout this whole sequence contrastive work is being undertaken that appears to be working to depict the complainant(s) as being polite, formal and reasonable versus the allegedly unreasonable and unprofessional behaviour of the complained about party - X.

#### Narrative objective or subjective?

All complaints evidenced clear tensions between objective and subjective accounts as the complainant revisited and relived the perceived transgressions and therefore, struggled to be, or remain an objective witness. Accordingly, discursive features become more pronounced as the complainants’ attempt to fully explicate the accumulating grievance (Table 2).

**Table 2:** the use of discursive features

Discursive Features	Example	ID
Capitalisation/bold/Exclamation	*BREAKDOWN IN COMMUNICATION!*	3948
	*We as a family want EVERY avenue explored.*	3946
	*There is much more*	3986
Active Voicing/reported speech	‘before you fall down drunk’	3965
Repetition	*‘my mum’* (39 times)	4002
	*‘traumatic journey’*	3932
Extreme Case Formulations (ECF)	*‘utterly appalled’* *extremely upsetting; great deal of stress; excruciating back pain;*	3986
*Superiority Humour: sarcasm*	*“found two staff members at the X reception desk, were apparently, honours graduates of the Stalinist School of Human Relations.”*	3952
*Idioms*	*‘It frightens me to think what would* *happen if my mum weren’t “on the ball”!*	3928
Questioning	*Why did X not phone from house* *At what time did X get back to X* *At what time did she speak to the Doctor* *At what time did Doctor phone for an ambulance* [sic; no punctuation provided]	3945

There were some clear passages of ‘objective testimonials’ but these invariably evolved into inferred neglect narratives and then became more overtly subjective as outlined above. Moreover, it could also be argued that some subjective accounts were actually presented within an objective frame of reference e.g. date, time, place - from the outset. What is important here is that the complainants apparently attempt to remain objective, or at least present themselves as being objective and accordingly, genuine and legitimate complainers. When they become more subjective and subsequently lose objectivity, they turn to other persuasive strategies such as personalizing the complaint, legitimising the complaint and epistemic authority.

##### Personalising the complaint

On occasions, some complainants personalised the complaint, using it to act as bridge between the descriptive facts initially provided and the more illustrative and contrastive accounts that emerged as the case built momentum and corroboration. Complainants provided individual, biographical information about the aggrieved party; *“my mum had a strong heart and was a fighter” [4002]* in contrast with the relatively objective narrative previously outlined. Thus, the abstract becomes manifest;

*‘I attach a photograph – [you] can see he was back to some degree of normality at Christmas*’ [3970].

The above account derives from a son’s powerful complaint about his father being accorded a ‘Do Not Attempt Cardio Pulmonary Resuscitation’ (DNACPR) record without the family’s knowledge. The photograph included with the complaint – made via his local Member of the Scottish Parliament (MSP) - illustrates his father’s well-being after making a full recovery from his illness. It therefore, accentuates the gravity of the complaint with irrefutable evidence and simultaneously humanizes the issue.

### Legitimising the complaint

All complaints evidenced the need to establish the legitimacy of the complaint to varying degrees. A legitimate complaint and complainant is someone who can justifiably detail misdemeanors and convince the reader of the grievance (e.g. [11, 39]). This persuasive strategy emphasized discrete entities such as patient compliance, reasonable expectations of help, a social conscience, concern for others’ (individual) safety, a perception of repeated transgressions and despite all this, a remaining reluctance to complain.

As a pre-requisite to reporting a series of transgressions, many complainants sought to establish themselves as a rule-follower, a ‘good’ patient (see [32]) - someone who knows the rules, respects the rules and is compliant.

*I understand the hospital is very busy’ [3963].*

*‘I arrived in good time [for my appointment]’ [3952].*

*‘Both my wife and I are members of the Institute of Advanced Motorists and we parked the car in a safe position’… [3967].*

The hospital is a particular institutional setting with set roles and routines that enable vast swathes of patients to be treated efficiently and effectively. Although patients expect to be treated as individuals, the complainants implicitly recognized that their individual needs may be subordinate to the demands of the many, especially in a publicly funded service. Hence, it was important for the complainant to recognise the demands on the health service and their explicit individual obedience. Central to the complainant’s explicit rule-following was their reasonable expectation of help from this publicly funded service: a recognition of both rights and responsibilities.

*Dr is supposed to help. I think this is hospital policy “we are here to help” [CAPITALS: 3963].*

Sometimes these expectations of help were implicit rather than explicit and occasionally juxtaposed with compliant behaviour;

*‘I take all precautions when attending appointments so I therefore expect that my well-being by NHS [sic] to be high on their agenda’ [3975].*

When the complainants’ *individual* expectations were not met, this invariably morphed into a broader expectation of help, befitting the general public’s protective sentiments towards this long-standing welfare service. Complainants were therefore, expressing a social conscience for the greater good as also noted by Simmons and Brennan [51, 55]. On one level these utterances epitomized the public’s sense of guardianship of the NHS encompassing its public image, financial prudence and accountability;

*‘the quality of service that I have received from X reflects badly on the NHS as a whole’. [3968].*

*‘not a very efficient or cost effective use of NHS time and money I think’ (3979).*

*‘Can we have accountability for the actions or inactions of public servants who fail to provide a fair and reasonable service in the execution of their duties’? (3970).*

However, on another level, if these custodial expectations were not met, complainants were not averse to outlining the potential risk involved for the individual or others;

*‘How many people have already been allowed to die?’ (3970).*

The complaint’s legitimacy was further enhanced when the strength of the perceived and repeated transgressions fused with the complainer’s sense of social justice and a concomitant reluctance to malign a public service. This ‘reluctance’ was usually outlined at the outset; ‘*with much regret’* (3979) and, or reiterated at the conclusion of the complaint:

*‘my strength of feeling has left (me) with no alternative’ (but to complain)’ (3979).*

It is important to appreciate the extent of legitimacy work evident in the complaints. The complainant does not need to justify their complaint yet, is clearly at pains to provide a strong foundation for the complaint and dismiss any suggestion that their complaint is vexatious.

### Epistemic authority - corroborating

To all intents and purposes, the weakness in any complaint is the potentially limited knowledge of the complainant with regard to medical conditions, interventions or the workings of healthcare systems and processes. This may arguably result in epistemic asymmetry with the potential to constrain any challenge to the perceived injustice(s) on the part of the complainant. Consequently, complainants sought to counterbalance this by various means with reference to knowledge proactively gleaned from different sources including the internet, carer experience, an epistemic friend (doctor, nurse) and, or the actions or comments of other healthcare professionals involved in the complaint.

*“While on the web we discovered” [the opposite treatment to that advocated]. [3946].*

*“All the other professionals involved appear to have the same view that she was very ill and needed immediate admission and treatment.” [3921].*

*“Her oxygen level was 88%, my understanding is that anything below 93% is concerning.” [4002].*

*“I work with X patients and would never….” [3965].*

The complainants therefore, assiduously used those formal and informal sources of information to simultaneously corroborate their case and offset an alternative dis-preferred account.

Within any epistemic relationship, specifically that between healthcare professional and patient, is an inequality of knowledge, a power imbalance and the potential for communicative constraints (e.g. [26]). The complaints demonstrated that irrespective of the rights and wrongs of the perceived injustice, it is clear that the reported interactions were unsuccessful and thus, the epistemic relationship had broken down. There were numerous examples of complainants providing what they felt was relevant and important information only to be met with responses which failed to evince the desired testimonial sensitivities.

## Discussion

The NHS provides life-saving and sustaining care and treatment to vulnerable, dependent individuals and their families. This intimate and fundamental service carries patients’ and their families’ hopes and expectations at difficult times. Abrogation of this responsibility understandably magnifies any perceived injustice. An online public survey (*n* = 4236) conducted by yougov noted that 90% believed people should complain about public services but of the 27% disaffected with public services in the past twelve months only 34% actually did so [42, 43]. Thus, complaints are representative of greater disaffection [3, 60]. The oft-stated aim of public service complaints is to prevent the same problem occurring again [6, 51, 55] with an explanation, apology [3] and accountability for actions also held dear [6]. Only one complainant in our data specified litigation as a possible option in keeping with the less litigious legal system in Scotland [52] and the enduring appeal of the NHS. All other complainants specified the desire for an investigation and explanation or, alternatively, requested a specific response to negatively formatted question(s) [33]. They therefore, sought understanding rather than recompense.

The typology of complainants in our data is similar to that reported elsewhere [35, 36, 46]. Moreover, irrespective of possible criticisms of our broad taxonomy, the focus, nature and outcome of complaints is also broadly similar to that elsewhere, with many being complex, subjective, emotive and unlikely to be upheld [44]. Although our study reviews direct as opposed to indirect interactional complaints, our data shares much in common with Conversational Analytic studies of complaints. For example, our sub-categories of legitimacy and epistemic authority [21] resonate with complaining as an accountable activity and the defensive implicit and explicit moral work evidenced elsewhere [2, 9, 11, 53]. Accordingly, our complainants worked hard not to be viewed as ungrateful or ‘dispositional moaners’ ([11]:4).

What is fascinating about our data is the amount of work, time and effort that has clearly gone into ‘building a case’ to convince the reader of the injustice and associated hurt. The complainants patently start out to build an objective case but this wanes as the defensive detailing ([8]:297) prompts the complainant to revisit the cumulative grievances and reveal an increasing sense of disempowerment.

Many of our complainants demonstrably found their experiences and those of their loved ones to be a traumatic event or events. In turn, their apparent repeated failure to arrest the cumulating grievance potentially also added to their trauma and sense of disempowerment. Caregiver psychological distress per se is well established e.g. [37]. However, it is interesting to note that psychological effects are likely to be most severe if trauma evidences, any or all of the following; is human-caused, repeated, unpredictable, multi-faceted, perpetrated by a caregiver, sadistic and undergone in childhood [22]. Only one, possibly two aspects on that list (sadistic, undergone in childhood) would not be relevant to most or all of the complex care and treatment complaints in our data corpus.

Our data evidenced complaints as individual narratives predicated on a moral imperative but laced with cynicism as demonstrated by the use of sarcasm, epistemic authority and the attempts at corroboration. Complainants reported cumulative attempts to resolve their grievances prior to submitting a formal complaint and the subsequent time and effort devoted to filing the complaint arguably corroborates the view that there is a lack of impartiality in the system [13], with transparent resolution unlikely [60] and an ongoing disaffection with the complaint handling process and outcome [13, 15]. In attempting to build a case, they try to obtain an explanation and some kind of redress for the hurt experienced, but in so doing are faced with reliving the trauma again and further, in committing it to print. Despite all that, it says something about the complainants in our data corpus, the level of hurt accrued and the concomitant sense of injustice about a publicly funded service, that they still expended considerable energy in trying to seek some resolution.

It is relatively unremarkable that, what people complain about is directly related to the way in which the complaint is expressed e.g. discursive features, legitimacy, epistemic authority and the amount of complex content. Perhaps, the complainants are, to all and intents and purposes, attempting to privately work their way through some kind of catharsis whilst remaining publicly strong and assertive (e.g. positive thinking; [31])? All the while they probably know that they are unlikely to achieve a positive resolution and hence, any attempt at objectivity and non-emotive discourse is a forlorn hope. Ironically, this associated ‘noise’ may well work against the veracity of the complainant’s case and mitigate a successful outcome. A further paper will highlight the lack of sensitivity and focus in the complaint handlers’ responses and note that the more complex and emotive complaints are less likely to be upheld. There is arguably a need for patients’ formulations of these grievances to be better understood and addressed more sensitively – even when a complaint is not upheld. Enhancing complaint handlers’ awareness of the rhetoric of complaints could facilitate a more appropriate and sensitive response. Importantly, it may also assist in facilitating the recovery of the complainant.

### Limitations

This paper outlines the characteristics of our written complaints’ data corpus and an overview of complainants’ accounts of their experiences or those of their loved ones and the rhetorical and persuasive strategies they employ. It does not provide information on the veracity or otherwise of the accounts and focuses only on the accounts of the complainants. The type of data (complaints only) and the specific type of data (written complaints only) are also limitations. Nevertheless, we have obtained a relatively large heterogenous data corpus in qualitative terms.

## Conclusion

Complaints are unhappy and distressing events for all concerned: patient, complainant, complaint handlers and staff. Bourne [5] recently noted significant psychological distress among complained-about doctors and highlighted their need for emotional support during the complaint handling process. Patients and their relatives have access to advocacy/support agencies when making a complaint. However, there is perhaps a need to proactively provide or offer emotional support or counselling and address their evident psychological distress. The NHS is expected to learn from negative feedback and facilitated feedback on complaints has been shown to have an impact on quality improvement [45]. However, all staff could benefit from better understanding how complaints are formulated and the ongoing psychological distress it provokes. This could also potentially, have an impact upon retention as complaints have been linked to low job satisfaction [23].

Despite the plethora of complaint management resolution guides and reviews (e.g. [38, 43, 47, 48]) healthcare complaints still evidence a lack of transparency [13] and appropriate resolution [14, 16]. We believe that all healthcare staff and complaint handlers in particular could benefit from a more patient-centered approach to complaints. A better understanding of the nuances of how patients formulate complaints should enable healthcare staff to respond more sensitively and appropriately to the complainants’ evident psychological distress. It may also help complaint handlers to focus in on the genuine concerns being raised as opposed to the ‘noise’ of the complaint. Complaint handlers may therefore, be better able to produce a more transparent and appropriate redress. In turn, this may help in reducing the harm, residual bitterness and lack of trust from unresolved complaints.

## Abbreviations

CH: Complaint Handler
DA: Discourse Analysis
DNACPR: Do Not Attempt Cardio Pulmonary Resuscitation
ECFs: Extreme Case Formulation
ESRC: Economic and Social Research Council
HSCIC: Health and Social Care Information Centre
NHS: National Health Service
NOK: Next of Kin
PHSO: Public Health Service Ombudsman
SPSO: Scottish Public Services Ombudsman
TV: Television
UK: United Kingdom (of Great Britain and Northern Ireland
